# Application of PMMA bone cement composited with bone-mineralized collagen in percutaneous kyphoplasty

**DOI:** 10.1093/rb/rbx019

**Published:** 2017-08-04

**Authors:** Ming Bai, Heping Yin, Jian Zhao, Yang Li, Yongdong Yang, Yimin Wu

**Affiliations:** 1 Department of Minimally Invasive Spinal Surgery of the Second Affiliated Hospital of Inner Mongolia Medical University, Hohhot 010030, China; 2 Inner Mongolia Medical University, Hohhot 010030, China; 3 Institute for Regenerative Biomaterials, Tsinghua University, Beijing 100084, China

**Keywords:** percutaneous kyphoplasty, osteoporotic vertebral compression fractures, bone cement, mineralized collagen

## Abstract

We investigated the feasibility of applying polymethylmethacrylate bone cement composited with biomimetic bone-mineralizsed collagen to percutaneous kyphoplasty (PKP). We performed PKP in 95 patients diagnosed with osteoporotic vertebral compression fracture. All patients had fractures of a single vertebral body, and they were divided randomly into control (group A, 47 patients) and experimental (group B, 48 patients) groups. Patients in group A were treated with acrylic cement, and those in group B were treated with acrylic cement composited with the bone graft material. All patients were evaluated by a visual analogue scale (VAS), Oswestry disability index (ODI), Cobb angle and anterior vertebral body height preoperatively, and 3 days and 3 months postoperatively. All patients successfully completed surgery and were followed up thereafter. The VAS score, ODI index, Cobb angle and anterior vertebral body height compression rate in both groups had significant changes (*P* < 0.05) preoperatively, and at 3 days and 3 months postoperatively. There was no significant difference between the two groups at different times (*P* > 0.05). The analgesic effects of bone cement composited with bone-mineralized collagen are similar to those of bone cement only. Mineralized collagen has excellent promotion prospects by inducing new bone formation and reducing the incidence of adverse reactions caused by bone cement.

## Introduction

Osteoporosis is a fracture-prone systemic disease characterized by osteopenia and bone mass losses. It is a progressive disease and a worldwide health problem that affects the quality of life of patients. The incidence of osteoporosis also increases with increasing age, and the incidence of osteoporotic vertebral compression fractures is ∼20% in postmenopausal women. Approximately one in three patients suffers from chronic pain in the waist and back. This may be because a humpback deformity may cause the chest to become smaller, which affects heart and lung functions to varying degrees. Osteoporosis, combined with low back pain and other symptoms, seriously affects quality of life and has become a major health problem for the elderly, especially for older women. Currently, the ageing population grown significantly, which has contributed to a heavy economic burden to society and families, and has aroused the attention of the medical community and society in general. How to improve the cure rate and reduce low back pain and other sequelae have become key problems in the treatment of osteoporotic thoracolumbar compression fractures [1–3]. Traditional therapies include bedrest, oral administration of calcium and analgesics, wearing a brace, physiotherapy, rehabilitation therapy and so forth, which has led to a sharp decline in the quality of life of patients and increased mortality.

Percutaneous vertebroplasty (PVP) is a newly developed treatment consisting of injecting filling materials into a loose vertebral body. A large number of surgical cases worldwide have proved PVP to be a simple, safe and effective minimally invasive procedure. However, although it can achieve quick alleviation of pain caused by the diseased vertebra, it cannot restore the vertebral height and cannot correct the kyphotic deformity of the vertebral body. Therefore, percutaneous kyphoplasty (PKP) has emerged as a new minimally invasive technique for spinal surgery in which a balloon is injected percutaneously primarily into the vertebral body through the pedicle of the vertebral arch, followed by an injection of filling materials (such as bone cement). These fillers not only can enhance the strength of the vertebral body but can also restore the vertebral height. Such procedures can achieve immediate stability and obvious relief of pain. Patients can get out of bed within 24 h and perform normal daily activities. In addition, the fillers can prevent a collapse of the injured vertebra, reconstruct the physiological curvature of the spine and restore the normal negative gravity line of the spine. The merits of this procedure include less trauma and good analgesic effects, and the rate of pain relief for osteoporotic vertebral compression fractures is estimated to be >90% [4].

The most common filler material is bone cement, which has good analgesic effectiveness. However, it also has many defects, such as poor biocompatibility, osteogenic inaction, deficiency in biodegradability and foreign body reaction on the bone cement surface, which may cause delayed bone resorption and finally lead to a decrease in the mechanical strength of the vertebrae. In this study, we compared the clinical effect of bone cement alone applied during PKP against that of bone cement composited with bone-mineralized collagen. The latter achieved satisfactory results, which we have reported.

## Materials and methods

### General information

A prospective analysis was performed on 95 patients (39 males and 56 females) who underwent PKP at the Department of Minimally Invasive Spinal Surgery of the Second Affiliated Hospital of Inner Mongolia Medical University from October 2013 to November 2015. They all suffered a single vertebral fracture, including fracture of the T11, T12, L1 and L2 vertebra bodies in 20, 28, 29 and 18 cases, respectively. The patients were randomly divided into two groups. Group 1 (control group) included 47 patients (20 males and 27 females, 51–76 years old; average age 56.2 ± 6.1 years). Ten patients had T11, 14 had T12, 14 had L1 and 9 had L2 fractures. Bone cement was injected into the diseased vertebrae. Group B (experimental group) included 48 patients (22 males and 26 females, 48–75 years old; average age 55.3 ± 7.1 years). In this group, 13 patients had T11, 14 had T2, 16 had L1 and 5 had L2 fractures. Conventional PKP was performed after a part of the bone cement was replaced with an equal amount of artificial bone-mineralized collagen.

Lumbar magnetic resonance imaging (MRI) examination was performed in all patients to confirm the diagnosis, and bone density examination showed osteoporosis. There was no sign or symptom of spinal cord or nerve root injuries preoperatively. Computed tomographic (CT) examinations did not show vertebral posterior wall damage, while X-ray examination revealed <50% of vertebral collapse. All patients felt pain in their back, particularly more intensely when they bore weight on the spine, turned over or sat up, before the operation. There was no statistical significance in sex, age and health condition between the two groups (*P *> 0.05).

### Surgical methods

The patients in Group A lay prone with their hipbones and knees bent with a cushion under their chest and hip, respectively, under conventional electrocardiographic monitoring by the anaesthetist. Conventional disinfection was performed with the puncture point of the core. Fractured vertebral pedicle surface projection and labelling were located fluoroscopically via a G-arm X-ray machine. Lidocaine was adopted for local anaesthesia. The vertebral pedicle was punctured in the outer upper corner (the left side was punctured at roughly the 10 o’clock position, while the right side was punctured at roughly the 2 o’clock position). Then, the vertebral centre was punctured along the bipedicular approach. The needle point was located on the inner margin of the vertebral pedicle as shown from the anteroposterior projection and passed through the vertebral posterior margin as shown from the lateral projection. The core of the puncture needle was pulled out with a working cannula indwelling. A manual bone drill was inserted into the puncture and then pulled out. A balloon filled with a contrast agent was inserted, and the pressurizer was pushed inward to read the digital pressure gauge. The balloon was expanded gradually under anteroposterior and lateral G-arm X-ray monitoring and decompressed after the fractured vertebrae were reduced. Afterwards, bone cement was prepared to a pasty consistency and 2–4 ml was pushed inwards. Intermittent observation was performed through the G-arm X-ray machine to confirm that the bone cement did not leak from the vertebral posterior margin. The working cannula was pulled out, haemostasis was achieved with local compression and a sterile dressing was applied.

The same surgical method was adopted for Group B. While the bone cement was being prepared, variant bone-mineralized collagen was poured into a mixing bowl first. An equal amount of bone cement was removed from the prepared cement, and the remaining bone cement was mixed with the variant bone-mineralized collagen and stirred. When the mixture became pasty, it was injected into the diseased vertebrae using the unfolder. Haemostasis was achieved with local compression and a sterile dressing was applied.

The bone cement used in this study was Osteopal polymethylmethacrylate (PMMA) (Heraeus, Germany) and the bone-mineralized collagen was manufactured by Beijing Allgens Medical Science and Technology Co. Ltd. The main compositions of the bone-mineralized collagen included hydroxyapatite (HA) and type-I collagen, which make up bionic bone tissue material. The bone-mineralized collagen is first poured a mixing bowl. Then, an equal amount of prepared bone cement is removed, and finally the remaining bone cement is mixed with the bone-mineralized collagen to prepare an implant material [5–7]. The ratio of bone cement to bone-mineralized collagen is 6:1.

### Postoperative treatment

The patients lay on their side for 2 h after the operation, so as to avoid choking should the bone cement reaction cause vomiting. They were allowed to ambulate on the floor 24 h later under the protection of a back pad. Calcitriol soft gelatin capsules and alendronate were administered to protect against bone loss.

### Observation targets

Vital signs were monitored continuously during the operation. Plain (anteroposterior and lateral) radiographs were taken 3 days postoperatively, and CT scanning of the diseased vertebrae was performed 3 months postoperatively to observe the distribution of the filler material in the vertebrae, the recovered height of the injured vertebrae, the status of leakage and the absorption state of bone-mineralized collagen in the vertebrae. Follow-up visits were conducted before the operation, and at 3 days and 3 months postoperatively for a statistical survey according to the pain visual analogue scale (VAS), Oswestry disability index (ODI), Cobb’s angle and anterior vertebral body height (AH = normal anterior flange height − anterior flange height after compression)/normal anterior flange height × 100%) compressibility evaluation. A paired *t*-test was conducted on the data, showing a statistically significant difference (*P *< 0.05).

## Results

Surgery was successful in all patients, and ∼2–4 ml of bone cement was injected into each vertebra (average 3.1 ± 1.0 ml). The duration of a single vertebral operation was ∼15–35 min (average 25 ± 3 min). The average amount of haemorrhage during a single vertebral operation was ∼10 ml. Group A received bone cement alone, while Group B received bone cement composited with bone-mineralized collagen. All patients were followed up for ∼9–16 months (average 13 ± 2 months). Images obtained at 3 months postoperatively were not significantly different from those obtained at 16 months. In both groups, back pain was significantly relieved postoperatively, as shown by VAS and ODI scores. At the same time, Cobb’s angle and anterior vertebral body height compressibility were improved (*P* < 0.05; Tables 1–4).

**Table 1:** Preoperative and postoperative back pain evaluation by VAS

Group	Preoperative	3 days postoperatively	3 months postoperatively
A	8.75±2.11	3.77±1.14*	1.98±1.39*
B	8.45±1.90	3.24±1.15*	1.52±1.15*
*t* value	0.77	2.41	1.99
*P* value	0.69	0.02	0.05

* *P *< 0.05 as shown by intragroup preoperative and postoperative comparison.

**Table 2:** Preoperative and postoperative scoring by ODI

Group	Preoperative	3 days postoperatively	3 months postoperatively
A	62.65±12.22	26.48±4.98*	16.95±13.23*
B	63.45 ± 11.91	25.24±5.14*	15.46±12.33*
*t* value	1.67	2.67	2.03
*P* value	0.79	0.04	0.05

* *P *< 0.05 as shown by intragroup preoperative and postoperative comparison.

**Table 3:** Preoperative and postoperative comparison of Cobb’s angle between groups A and B

**Group**	Preoperative	3 days postoperatively	3 months postoperatively
A	19.7±2.14	3.6±1.10*	4.2±1.33*
B	19.4±1.94	3.1±1.25*	3.7±1.25*
*t* value	0.66	2.25	2.15
*P* value	0.44	0.03	0.03

* *P *< 0.05 as shown by intragroup preoperative and postoperative comparison.

**Table 4:** Preoperative and postoperative comparison of anterior vertebral body height compressibility evaluation between groups A and B

Group	Preoperative	3 days postoperatively	3 months postoperatively
A	58.2±6.73	5.3±1.85*	6.1±1.90*
B	57.8±7.42	5.9±1.71*	6.7±1.82*
*t* value	0.73	2.19	2.14
*P* value	0.04	0.03	0.03

* *P *< 0.05 as shown by intragroup preoperative and postoperative comparison.

**Figure 1:**
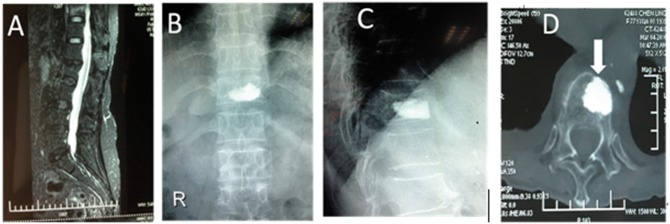
A 77-year-old patient underwent PKP for T11 osteoporotic vertebral compression fractures. (**A**) Preoperative MRI shows the compression fracture of the T11 vertebral body. High signal intensity is shown in the fat-suppression image. (**B, C**) Anteroposterior and lateral projection re-examination performed 3 days postoperatively. (**D**) CT scan performed 3 months postoperatively shows a clear boundary between the bone cement and bone tissues (arrow).

**Figure 2:**
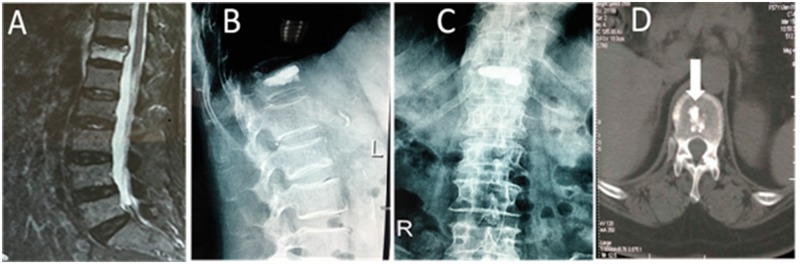
A 57-year-old patient underwent PKP for T12 osteoporotic vertebral compression fractures. (**A**) Preoperative MRI shows the compression fracture of theT12 vertebral body. High signal intensity is shown in the fat-suppression image. (**B, C**) Anteroposterior and lateral radiographs performed 3 days postoperatively. (**D**) CT scan 3 months postoperatively shows a fuzzy boundary between the bone cement composited with bone-mineralized collagen and the bone tissues around it (arrow). It is clearly seen that part of the bone cement is already substituted with new bone tissues.

X-ray re-examination 3 days postoperatively showed that the filler material was in good condition and the compressed vertebrae had recovered to varying height. There was no complication, such as pulmonary embolism, infection or nerve injury. A satisfactory curative effect was achieved in all patients. CT re-examination at 3 months postoperatively showed that the bone cement was still clearly visible in Group A, and there was a clear boundary between the bone cement and the vertebral bone tissues (Fig. 1). The bone cement composited with bone-mineralized collagen was visible among vertebral bone tissues, but the boundary was not clear, and bone substitution was creeping between the bones (Fig. 2).

## Discussion

In recent years, PVP and PKP have been performed worldwide. Owing to their characteristics, such as minimal invasiveness, high relief of pain caused by osteoporotic vertebral fractures and high safety, they have become important methods to treat osteoporotic thoracolumbar compression fractures, vertebral metastases, vertebral haemangioma, myelomas and other relevant vertebral diseases.

PVP and PKP are technologies used primarily to inject fillers into diseased vertebrae, to stabilize vertebrae, and to relieve pain based on the heat released when fillers strengthen and polymerize vertebrae. There are a great variety of fillers, such as PMMA, calcium phosphate cement (CPC), calcium sulphate cement (CSC) and some other common fillers. Up to 66% of complications occurring during PVP and 73% of complications occurring during PKP are concerned with the characteristics of bone cement. Since it releases heat (35–90°C) during coagulation, the peripheral tissues and nerves will be burned in case of leakage, causing significant complications. Due to its poor biocompatibility, degradability and absorbability, PMMA becomes a permanent foreign body once injected, and cannot form a chemical bond with the bone tissues around it. Therefore, mechanical instability occurs due to looseness between bone and bone cement with time. By then, the neighbouring vertebrae will have suffered a secondary fracture. The PMMA macromonomer has cytotoxicity to vascular endothelial cells, which promotes thrombogenesis; therefore, its reflux with the vein after extravasation may cause significant complications [8–10], such as pulmonary embolism, myocardial infarction, cerebral infarction and decreased blood pressure, which may greatly affect elderly patients. Due to the absence of osteoconduction, PMMA cannot connect to natural bone. Due to its short bioactivity, it cannot induce new bone formation. Postoperatively, the mechanical strength of the diseased vertebrae will be different from that of the surrounding vertebrae. In particular, there will be an excessive difference in strength and stiffness between augmented and adjacent level vertebrae, which will increase the stress on adjacent vertebrae and make them susceptible to fracturing [11–14]. Mechanically, the key role of bone cement is to restore vertebral height, stabilize vertebrae and offer mechanical support to diseased vertebrae [15]. However, bone cement is not a glue. Thus, it cannot serve as an adhesive to form a chemical bond with diseased vertebrae [16]. Instead, it may loosen or move in the diseased vertebrae, and even move out of the diseased vertebrae into the spinal canal or abdominal cavity, causing serious consequences. At present, the most widely discussed and most commonly used bone cement substitutes are injectable CSC and CPC, both of which can be good solutions to the aforementioned shortcomings of bone cement. For example, the heat of polymerization is low at solidification (<40°C). Neither material will damage the surrounding tissues and nerves even if leakage occurs. Their tissue compatibility is good, and they can be degraded and absorbed completely. Both will strongly induce new bone formation and will not become permanent foreign bodies. Complete absorption generally can be achieved ∼12 weeks after injection. These materials have no cytotoxicity, do not damage vascular endothelial cells and do not cause serious complications, such as pulmonary embolism, myocardial infarction, cerebral infarction and blood pressure decline. Their mechanical strength is comparable to that of cancellous bone after hardening, and their application can avoid secondary fracture of adjacent vertebral bodies. However, they also have some disadvantages, including poor developing properties. They are difficult to inject and manipulate surgically compared to bone cement. Their absorption is faster, and their compressive strength is lower than that of vertebral cancellous bone. Accompanied by the possibility of vertebral height loss, their analgesic effect is inferior to that of bone cement. All the aforementioned materials have their own shortcomings and cannot completely meet the needs of repair of the fractured vertebral body. To this end, according to the characteristics of various materials and the needs of the clinical bone defect repair, different materials were combined in our experiment, so that the defects of the various filling materials were avoided and satisfactory results were eventually obtained.

Clinicians and scientific researchers have sought an ideal filler material. The study of bionic bone repair composite is helpful to the research of bone implants. Different materials were once composited together in the past, such as polylactic acid and HA composited together to prepare a bone-mineralized collagen composite that could promote bone repair with weaknesses offset by strengths [17, 18]. Chinese scholars Cui and Qiu [19–22] conducted *in vitro* animal tests proving that bone cement composited with bone-mineralized collagen could obviously improve the elastic modulus of bone cement. With time, new bone tissues would grow gradually into the porous structure to take shape after absorption of the bone-mineralized collagen.

An ideal type of modified bone cement should not only be operable, injectable and mechanically strong in clinical practice but also reduce its own elastic modulus after solidification, enhance its own biocompatibility to improve the stress distribution in diseased vertebrae and prevent itself from damaging surrounding tissues. Moreover, it should be able to integrate itself into the autogenous bone tissues in patients and fasten itself to diseased vertebrae steadily. Thus, it is a good choice to mix bone cement with a bone-mineralized collagen. The adulteration of a bone-mineralized collagen in bone cement not only can retain the advantages of bone cement, but can also overcome its shortcomings, such as non-degradability and non-absorbability, so as to improve its mechanical performance and biocompatibility.

In this study, by adding a bone-mineralized collagen to bone cement, we reduced its compressive elastic modulus while its solidification intensity remained the same, lowered the stiffness of diseased vertebrae, effectively prevented it from abrading the surface of diseased vertebral bone tissues and reduced the risk of secondary fracture in the adjacent vertebrae. Moreover, CT re-examinations at 3 months postoperatively showed that the bone-mineralized collagen could significantly improve the biocompatibility of the bone cement, attach bone tissues to the bone cement to grow on it, guide creeping substitution of new bone tissues, enhance the integration ability of the bone cement in diseased vertebrae and effectively protect against loosening and even detachment of the bone cement in diseased vertebrae. Evidently, the rate of adverse reaction caused by the bone cement was lowered, and its safety and long-term effects were strengthened. In addition, clinical practice has proven that bone-mineralized collagen has little impact on the injectability and operability of bone cement, suggesting that it meets the clinical requirements and has good prospects for application.

## Funding

This work is in part supported by NSFC No. 51402176, and the Inner Mongolia autonomous region natural science fund No. 20070501.

*Conflict of interest statement*. None declared.
